# Visual nudging of navigation strategies improves frequency discrimination during auditory-guided locomotion

**DOI:** 10.3389/fnins.2025.1535759

**Published:** 2025-03-19

**Authors:** Annalenia Malzacher, Tobias Hilbig, Michael Pecka, Dardo N. Ferreiro

**Affiliations:** ^1^Division of Neurobiology, Faculty of Biology, LMU Biocenter, Ludwig Maximilian University, Munich, Germany; ^2^TUM School of Life Sciences, Technical University of Munich, Freising-Weihenstephan, Germany; ^3^TUM School of Computation, Information and Technology, Technical University of Munich, Munich, Germany; ^4^Department of Computer Science and Mathematics, Munich University of Applied Sciences, Munich, Germany; ^5^Department of General Psychology and Education, Ludwig Maximilian University, Munich, Germany

**Keywords:** auditory perception, audiovisual interaction, active sensing, sensory nudging, multimodal integration, SITh, naturalistic experimental design, self-movement

## Abstract

Perception in natural environments requires integrating multisensory inputs while navigating our surroundings. During locomotion, sensory cues such as vision and audition change coherently, providing crucial environmental information. This integration may affect perceptual thresholds due to sensory interference. Vision often dominates in multimodal contexts, overshadowing auditory information and potentially degrading audition. While traditional laboratory experiments offer controlled insights into sensory integration, they often fail to replicate the dynamic, multisensory interactions of real-world behavior. We used a naturalistic paradigm in which participants navigate an arena searching for a target guided by position-dependent auditory cues. Previous findings showed that frequency discrimination thresholds during self-motion matched those in stationary paradigms, even though participants often relied on visually dominated navigation instead of auditory feedback. This suggested that vision might affect auditory perceptual thresholds in naturalistic settings. Here, we manipulated visual input to examine its effect on frequency discrimination and search strategy selection. By degrading visual input, we nudged participants’ attention toward audition, leveraging subtle sensory adjustments to promote adaptive use of auditory cues without restricting their freedom of choice. Thus, this approach explores how attentional shifts influence multisensory integration during self-motion. Our results show that frequency discrimination thresholds improved by restricting visual input, suggesting that reducing visual interference can increase auditory sensitivity. This is consistent with adaptive behavioral theories, suggesting that individuals can dynamically adjust their perceptual strategies to leverage the most reliable sensory inputs. These findings contribute to a better understanding of multisensory integration, highlighting the flexibility of sensory systems in complex environments.

## Introduction

A central goal of psychophysics is determining perceptual thresholds, which aims to reveal the fundamental mechanisms by which our senses operate (Link, 2001). Traditionally, experiments to assess perceptual capabilities are conducted in highly controlled laboratory settings in which sensory cues are carefully isolated. These controlled environments allow for the precise measurement of sensory thresholds by minimizing additional variables that could interfere with the specific sensory modality under investigation (Moore, 1973; Sek and Moore, 1995). Yet, while effective for isolating sensory processes, these stationary, simplified conditions differ significantly from the complexities of natural environments, which shaped the evolution of multimodal perception of sensory cues.

In real-world settings, sensory perception involves the integration of multisensory input as individuals actively navigate their surroundings (Soto-Faraco et al., 2019; Berthoz and Viaud-Delmon, 1999; Quak et al., 2015). During natural behavior, sensory cues from different modalities—such as vision and audition—often change coherently with self-motion, providing a dynamic flow of information about the environment (Chaudhary et al., 2022). This multisensory integration can have either beneficial effects, such as enhancing perceptual thresholds, or detrimental effects, where thresholds worsen due to sensory interference (Magnotti et al., 2018; Spence and Soto-Faraco, 2012). Particularly, vision often dominates in multimodal contexts, which may interfere with a participant’s ability to fully attend to other, e.g., auditory, information (Dietze and Poth, 2023; Posner et al., 1976). This phenomenon is highlighted by the Colavita visual dominance effect (Colavita, 1974), which occurs when participants fail to respond to auditory targets in the presence of simultaneous visual and auditory stimuli, even when explicitly instructed to do so. Research by Spence et al. (2012) corroborates this effect, showing that visual dominance can suppress awareness or responses to auditory input under these circumstances, underscoring the competitive dynamics between sensory modalities. Likewise, locomotion often favors reliance on visual information, which could impair auditory discrimination thresholds (Chen et al., 2017). However, it is still unclear how visual and auditory information are integrated in natural, dynamic environments involving self-motion.

**Figure 1:**
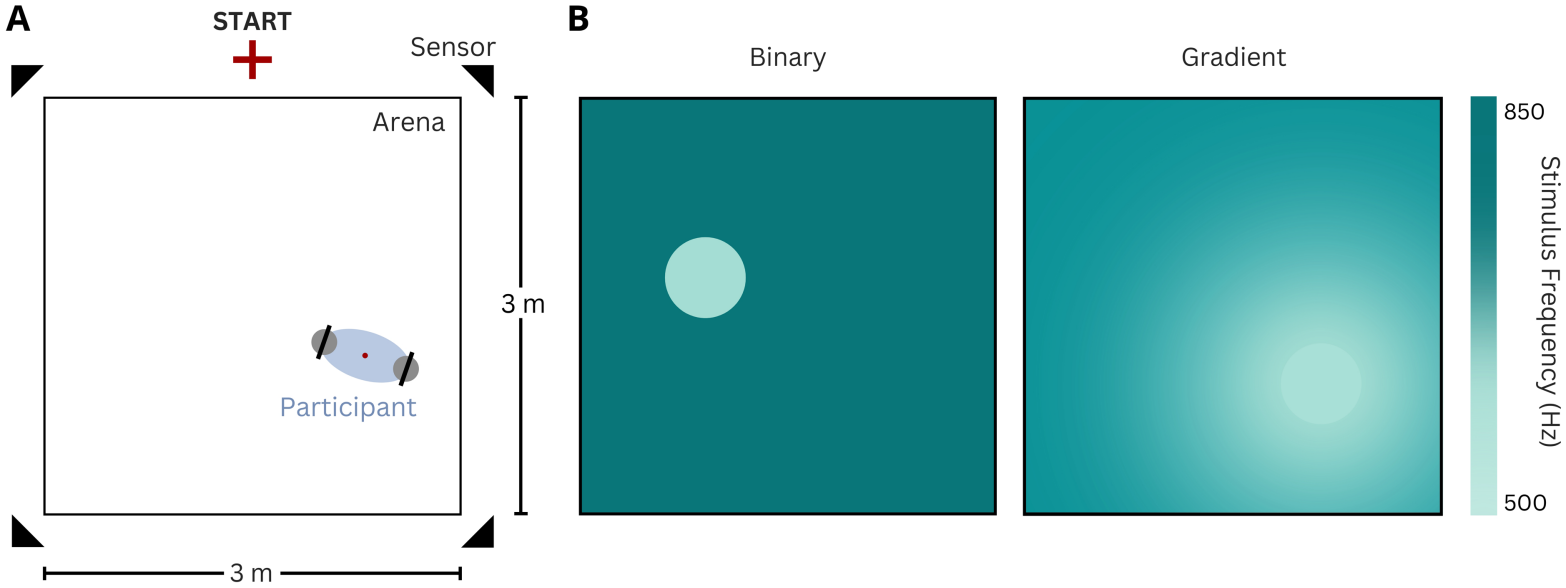
**(A)** Experiment set-up. The starting point of trials is depicted, as well as the four movement sensors, and the hand-held controllers. The red dot depicts the calculated online position of the participants by averaging the controllers’ position on the horizontal plane. **(B)** Illustration of the two task versions (left: binary, right: gradient). The colorbar illustrates the sound frequency played at the arena locations. The gradient encompassed the entire arena.

To promote the ethological significance of experimental paradigms, we previously developed the Sensory Island Task for humans (SITh; Ferreiro et al., 2022). SITh is designed to replicate natural behavior by allowing participants to engage in active, self-directed navigation while tracking an auditory target (Figure 1). Inspired by the classic ‘hide-and-seek’ scenario, participants search for an auditory “target island” within a 3 × 3 meter arena, guided by real-time changes in frequency cues modulated according to their position. Hence, participants are given the opportunity to use systematic frequency changes to navigate the arena and steer toward the target location. Using this task, we found that frequency discrimination thresholds during self-motion matched those reported in traditional, stationary paradigms, suggesting that naturalistic conditions do not inherently compromise auditory perception (Ferreiro et al., 2022). Given the reported dominance of visual information during navigational tasks (Colavita, 1974; Chen et al., 2017), this close match may be surprising. Specifically, we noticed that a large fraction of participants deviated strongly from an optimal search strategy that is directly based on self-motion-induced feedback in acoustic cues (“adaptive”), and instead patterned their search trajectory solely based on the arena geometry (i.e., visually dominated strategies, “non-adaptive”). Thus, the true perceptual limits under naturalistic conditions may be masked by visual interference and/or limited attentional capacity (Wahn and König, 2015). It remained unclear, however, whether participants’ perceptual thresholds can be improved by influencing their choice of strategy to solve a sensory discrimination task.

Building on these insights and the resulting questions, this study investigates how frequency discrimination thresholds in the SITh paradigm are affected when visual input is systematically altered. Specifically, by manipulating visual conditions, we clarify the impact of multisensory interactions during self-motion. In favor of maintaining a naturalistic experimental design, we induced an indirect shift in participants’ attention toward audition. For this purpose we degraded visual input, therefore nudging participants to focus more on audition. Nudges in choice architecture—such as structuring options or adjusting sensory cues—have been shown to guide behavior effectively without restricting freedom of choice (Thaler and Sunstein, 2008; Beshears and Kosowsky, 2020). A significant aspect of nudging lies in how attention is focused. Humans are boundedly rational, often making decisions based on information that is immediately accessible rather than focusing on relevant details (Simon, 1955). Thus, how information is presented — what we focus on — can influence choices (Kahneman, 2011). Nudges that structure decisions by organizing options help steer behavior effectively by lowering cognitive demands (Johnson and Goldstein, 2003). Sensory modalities such as vision and audition are integral to how we interpret information, and nudging individuals to focus on specific sensory cues may enhance perceptual decision accuracy. By drawing on the concept of nudging, we tested the effects of increased attention to audition, the corresponding impact on participants’ choice of search strategy, and determined how it affects their frequency discrimination thresholds.

Specifically, by incorporating visual manipulations, we explore how directing attention away from vision and/or toward audition affects frequency discrimination in an adaptive closed-loop self-motion paradigm, shedding light on the interplay between sensory inputs in a dynamic, naturalistic context. This approach contributes to a deeper understanding of sensory integration and perceptual decision-making under conditions that mimic real-world behavior.

## Methods

In this study, 16 normal hearing adults (8 female, 8 male) participated in two versions of the Sensory Island Task for Humans (SITh). All participants declared having no hearing problems. To ensure this, we conducted hearing tests to measure hearing thresholds between 125 Hz and 2000 Hz for each participant. Importantly, the tested range encompasses the stimulation frequencies used in this study.

### Setup and procedure

The experimental setup and general procedure were adopted as described in detail by Ferreiro et al. (2022). The experiment comprised two task versions and three visual conditions. Each participant completed 30 trials of each task version and visual condition.

Throughout all experiments, participants were free to move while searching for the invisible target island, which triggered the playback of the target tone upon entry. Three sound pips of the target frequency were presented to the participants before the start of every trial. Participants were instructed to search for a zone within the arena, in which the target frequency was presented. They were informed that in the binary task, the background tone in the arena would have a consistent frequency, while in the gradient task, the tone would vary in the form of a gradient approximating the target frequency based on the participant’s distance to the target zone (Figure 1). Importantly, they received no information about the shape or size of the zone, nor instructions on how to search, or feedback on their performance at any given point during the experiment. The target island (radius = 26 cm) was randomly positioned in each trial. With a surface area of approximately 0.212 m^2^, the island accounted for 2.36% of the total arena space.

Participants completed all trials for both task versions by freely navigating the physical arena. The starting point of every trial was fixed to a position 20 cm perpendicular from the center of one side of the square arena border and marked accordingly on the floor. Participants’ positions within the arena were continuously tracked using four infrared positional sensors (Oculus Rift S), while they held two Oculus Rift S controllers (Figure 1). The VR headset was not used in this study and was never worn by the participants, and therefore head position and movements were not recorded. The position data were collected at a rate of 20 Hz. Auditory stimuli were delivered through bluetooth headphones (Sennheiser 450 T). They consisted of 50 ms pure tone pips repeated every 250 ms (4 Hz repetition rate), and were ramped in and out using a 10 ms cosine window, with a 60 dB SPL amplitude, roved by +/− 5 dB (see task versions). Auditory feedback was always present, in all trials, and was only stopped if and when participants stepped out of the arena perimeter, and resumed as soon as their tracked position entered the arena again.

After having found the island within the arena, individuals had to press a button in the right-hand controller to end the trial. Alternatively, the trial was terminated automatically if the participant did not find and confirm the island within a time limit of 90 s.

### Task versions

All individuals first participated in the binary sensory island task, followed by the gradient sensory island task. Both task versions were completed under three different visual conditions each. The conditions were free vision (control condition, CC), distorted vision (nudging condition, NC: goggles with inserted prisms and blindfolded (blind condition, BC). When in the neutral position, the prism goggles flip the visual field vertically. The rotation of the prisms rotated the visual field independently for each eye 45 degrees, in opposite directions. These distortions produced the following effects on the visual input: horizontal head shifts moved the visual field upwards for one eye, and downwards for the other (depending on the direction of movement); and vertical head shifts moved the visual field either away or toward the midline, also depending on the direction of movement. Importantly, participants were instructed to keep their eyes open while wearing the prism goggles to guarantee that visual input remained present. All participants performed all experimental conditions in the same order. First, the binary task was performed, and then the gradient. Within the binary/gradient auditory task, participants performed first the CC, then the NC, and finally the BC visual condition.

In the binary task, the target frequency presented inside the island was 500 Hz, and the background frequency that was played in the rest of the arena was 850 Hz. In the gradient task, the tone presented at the target island was also set to 500 Hz. However, the background frequency in the rest of the arena varied in real-time based on the participant’s distance from the island and was defined by the following equation:

$\mathrm{Frequenc}y_{\mathrm{Gradient}}=\mathrm{Frequenc}y_{\mathrm{Target}}*1.001^{d}$

where *Frequency_Gradient_* refers to the frequency of the stimulus (in Hz) when the participant was outside the target island, *Frequency_Target_* is the 500 Hz tone presented within the island, and ‘d’ represents the participant’s distance from the island’s circumference in centimeters.

### Strategy classification

**Figure 2:**
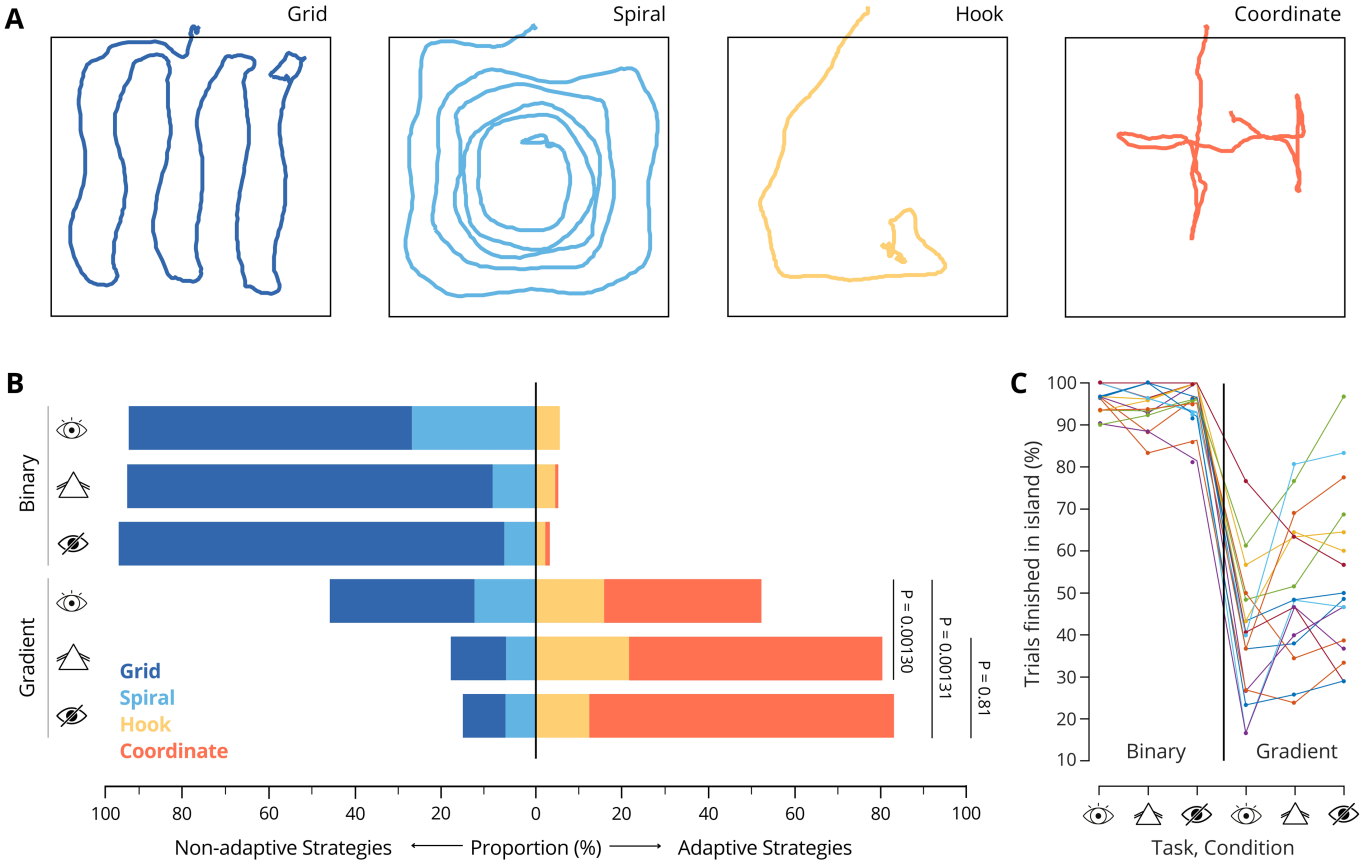
**(A)** Examples of the identified search strategy patterns. **(B)** Proportions of strategy usage across all participants and trials, by task. *p* values correspond to Wilcoxon signed-rank tests comparing proportions of adaptive trials, across participants: P_CC-NC_ = 1.30×10^−3^, P_CC-BC_ = 1.31×10^−3^, P_NC-BC_ = 0.81 **(C)** Percentage of trials completed within the island in each condition. Each line represents a participant.

Trajectories of walked paths were generated using Matlab for all trials. All images were analyzed visually by two unbiased individuals, completely naive to the study goals and methods, who received concise analysis instructions but did not participate in the experiment themselves and one individual with full knowledge about the study. The three evaluators classified trial trajectories independently, based on their shape. They categorized the trials either into the four identified strategies (grid, spiral, hook, coordinate; Figure 2A) or, if they could not identify any pattern, flagged these trials as uncategorizable. Images with a unique mixture of two or more of the four original strategies were assigned the most prominent one. After all the evaluators completed this, their assignments were compared, and the final classification of each trial was determined by a consensus criterion, in which at least two of them had to agree on the strategy used.

### Statistics

Friedman tests were conducted to assess differences across experimental conditions. Pairwise comparisons of distributions were performed using Wilcoxon signed-rank tests, and multiple comparisons were Bonferroni corrected. These non-parametric tests were selected because they are better suited for data distributions that deviate from normality, as is the case for our skewed data distributions. Normality of the Last Frequency Heard distributions was assessed with Anderson-Darling tests, with all conditions having a *p*-value <0.001.

## Results

In this study, sixteen normal-hearing young adults participated in the self-motion-based closed-loop SITh paradigm. Their task was to locate a target island within a 3 × 3 meter arena, allowing the determination of frequency discrimination thresholds for a pure tone stimulus and the examination of associated search strategies.

The target island was the sole location in the arena that elicited the playback of the target frequency (Figure 1). We examined two versions of SITh. In the binary version, the island can be easily distinguished from the non-target frequency (850 Hz) presented in the rest of the arena. In contrast, the gradient version was based on a radial frequency gradient across the arena, incorporating the frequencies from the binary task (Figure 1).

First, we investigated whether the participants employed specific strategies to search for the island and whether the choice of strategy influenced their performance in the two task versions. By analyzing the trajectories of all completed trials across participants (see Methods), we identified the same four distinct search strategies that we previously reported for SITh (Ferreiro et al., 2022). Two of these strategies are classified as non-adaptive—termed *grid* and *spiral*—while the other two are adaptive strategies, referred to as *hook* and *coordinate* (Figure 2A). The term *non-adaptive* refers to strategies that do not involve behavior adjustment to task-specific auditory cues. Instead, these strategies are shaped by the visually defined spatial structure of the environment (i.e., the experimental arena) to optimally cover the exploration area. In contrast, *adaptive* strategies involve behaviors that actively engage with the provided auditory feedback — namely, the frequency gradient - for maneuvering and guiding online navigational decisions, efficiently using the sensory information available.

While auditory feedback was present across all trials, we evaluated the participants’ performances for both task versions under three visual conditions:


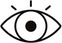
 control condition (CC): participants were allowed free, unaltered visual input.


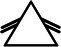
 nudge condition (NC): participants wore prism goggles that independently rotated the visual field for each eye (see Methods).


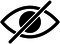
 blindfold condition (BC): participants wore completely dark blindfolds.

Our rationale for the nudge condition was that this visual degradation could shift attention away from the (non-task-relevant) visual information and toward auditory cues, which were required for successful task completion. This condition intended to nudge participants to prefer adaptive over non-adaptive strategies. For the blind condition, the lack of visual input should maximize the likelihood that participants would rely on the provided auditory cues and thus use adaptive strategies. This experimental design allowed us to assess the relative contribution of visual information to task performance, strategy choice and the resulting frequency discrimination thresholds.

Out of a total of 2,926 completed trials across all participants and conditions, only 166 trials timed out (0.06%). These include 144 in the binary task (6 in CC, 73 in NC, 65 in BC) and 22 trials in the gradient task (4 in CC, 4 in NC, 9 in BC). These numbers show that task completion rates were very high across all experimental conditions.

Participants used 4 different search strategies during task performance (Figure 2A). In the binary task, the *grid* strategy was the most frequently used across all visual conditions. Moreover, when visual input was impaired, the proportion of trials using this strategy increased further (Figure 2B; CC: 65.7%, NC: 85.1%, BC: 89.6%). The use of adaptive strategies remained marginal during NC and BC, which was expected, as no gradient information was present that could have been used for navigation even in the absence of meaningful visual input. Across participants, 96.1 ± 3.2% (mean ± SD) of all trials were completed within the target island during CC, which is consistent with our previous results (Ferreiro et al., 2022). Notably, performance remained near perfect when participants wore prism goggles (94.6 ± 4.8%) or were blindfolded (95.4 ± 5.4%).

In contrast, in the gradient task, adaptive strategies were generally favored across conditions. In CC, both non-adaptive and adaptive strategies were employed at similar rates (Figure 2B, CC: grid = 34.1%; spiral = 13.8%; hook = 15.6%; coordinate = 36.5%). Notably, when the participants’ vision was altered, there was a pronounced shift toward adaptive strategies, with the *coordinate* strategy becoming predominant (NC: grid = 12.5%; spiral = 6.9%; hook = 21.8%; coordinate = 58.8%. BC: grid = 10.2%; spiral = 7.0%; hook = 12.2%; coordinate = 70.6%). This effect was comparable in magnitude between NC and BC, suggesting that distorting visual input with the prism goggles was sufficient to nudge participants to rely more on auditory cues which correlated with their search strategy choice, in a comparable manner as without any visual input.

Notably, not only the fraction of adaptive strategies but also the proportion of trials completed in the target island increased for NC and BC compared with CC (Figures 2B,C), suggesting that the shift in strategy could be associated with changes in perceptual acuity. Thus, we next aimed to investigate to what extent the strategy shifts influenced performance.

**Figure 3:**
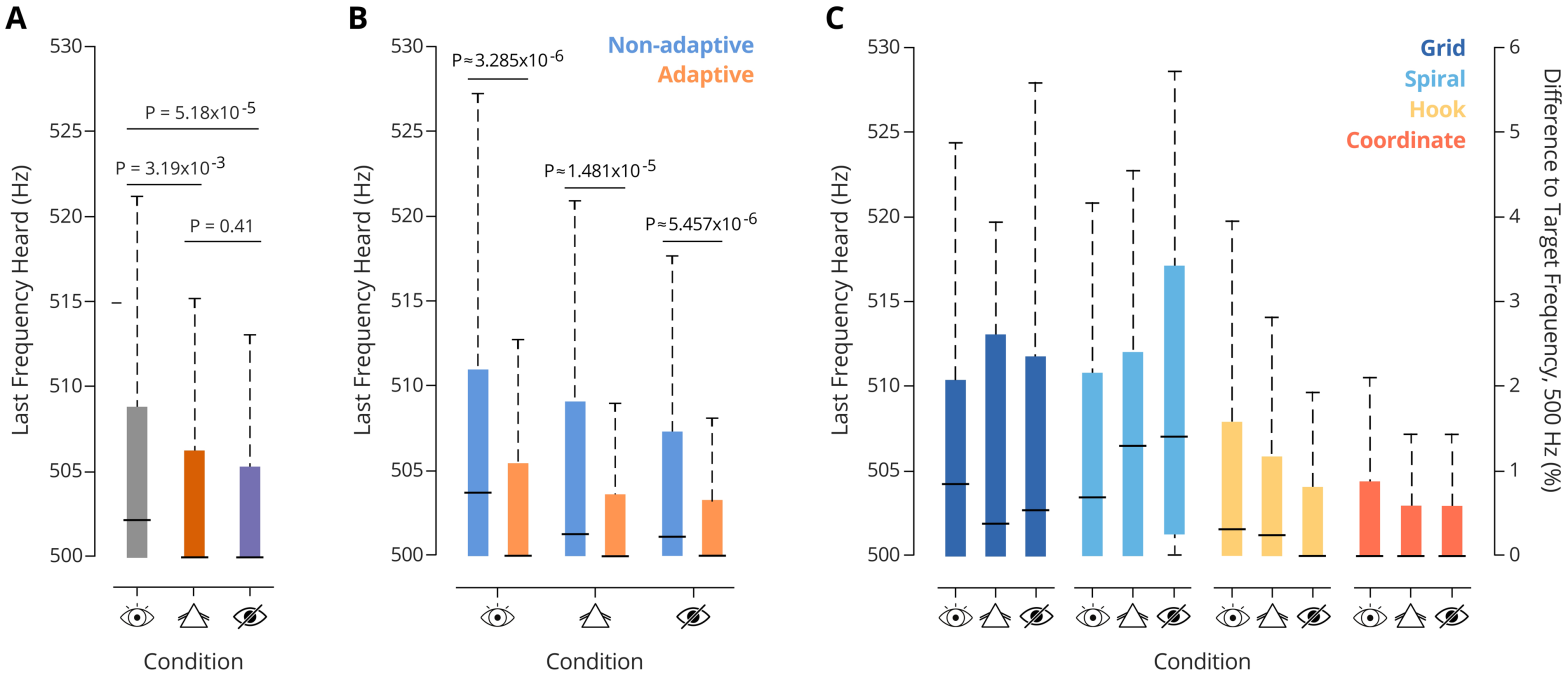
**(A)** Last Frequency heard at the end of trial, per task (Friedman test: *p* = 9.23×10^−7^. Pairwise comparisons correspond to Wilcoxon signed-ranktests: P_CC-NC_ = 3.19×10^−3^; P_CC-BC_ = 5.18×10^−5^; P_NC-BC_ = 0.41). **(B)** Last frequency heard, by strategy type, in the three different visual conditions for the gradient task. Notably, adaptive strategies elicited lower auditory thresholds. **(C)** Last frequency heard, by strategy, across tasks. Friedman tests: P_grid_ = 0.72, P_spiral_ = 0.68, P_hook_ = 0.18, P_coordinate_ = 0.79. Performance of each strategy was not different across visual conditions.

To this end, we first calculated the population discrimination thresholds for the different search strategies in the gradient task version by determining the last frequency heard (LFH) when finishing a trial. For this, the distance of the participant to the island border at the moment of button-pressing was used. Under all three visual conditions, performance was excellent (Figure 3A): The median LFH in CC was 502.25 Hz, consistent with prior data based on SITh (median 501.5 Hz), and reports using passive paradigms (Micheyl et al., 2012). Interestingly, on average, frequency discrimination thresholds further improved significantly under both visually restricted conditions (NC and BC).

**Figure 4:**
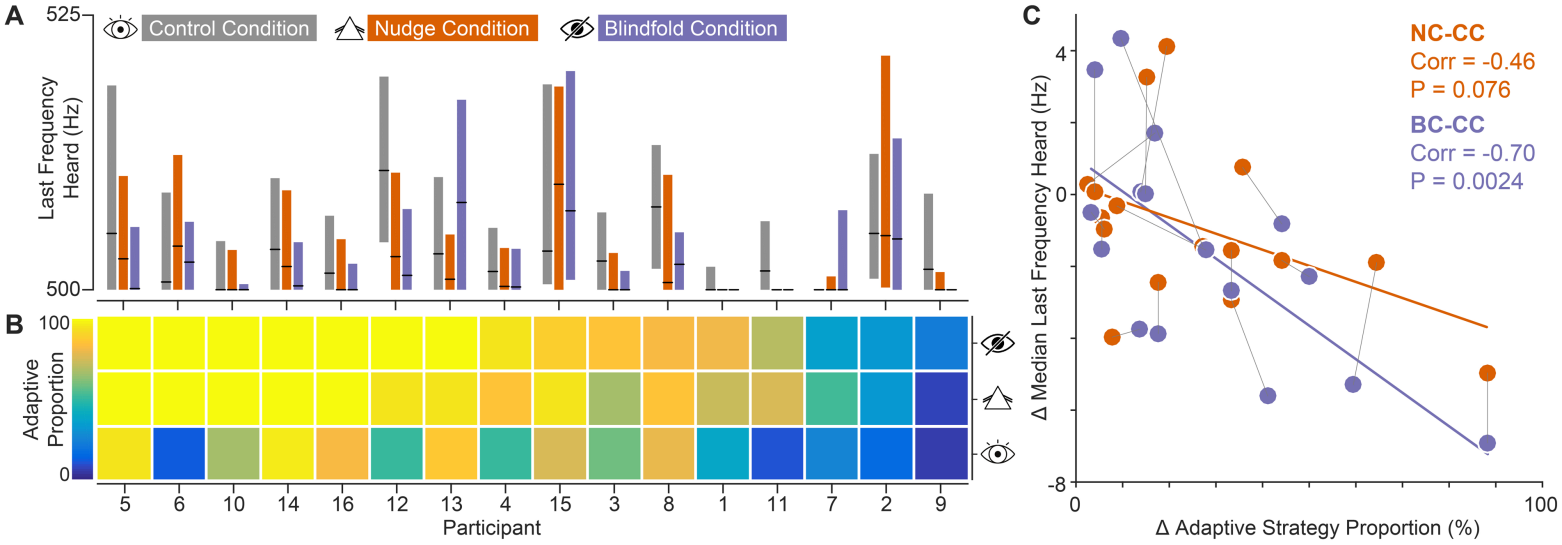
Individual participants’ data. Nudging participants to adopt adaptive search strategies improves auditory frequency discrimination thresholds. **(A)** Auditory discrimination performance across visual conditions. Black line markers represent the median. Boxes represent the interquartile range of the distribution. **(B)** Proportion of trials using adaptive strategies, across the three gradient visual conditions. Participants are ordered by descending proportions in BC trials. **(C)** Relationship between the change in adaptive strategy usage proportions and the change in auditory threshold. The displayed Pearson correlation coefficient (corr) reflects the strength of this association. Orange dots represent the change between NC and CC. Purple dots represent the change between BC and CC. Dots connected with thin black lines represent the same participant.

To investigate the extent to which this improvement was associated with the strategy choice, we compared the median LFH between trials in which participants used either *adaptive* or *non-adaptive* strategies. This analysis revealed significantly better thresholds for adaptive strategies under all three visual conditions (Figure 3B). Notably, the median LFH remained stable across visual conditions for each strategy (Friedman tests across visual conditions: P_hook_ = 0.18; P_grid_ = 0.72; P_spiral_ = 0.68; P_coordinate_ = 0.79). Thus, no influence of visual condition was observed on the auditory discrimination performance of the strategies (Figure 3C). Overall, adaptive strategies were strongly favored in NC and BC relative to CC (see Figure 2B for population data, and Figure 4A for individual data).

Together with the finding that participants shifted drastically from non-adaptive to adaptive strategies (Figure 2B), these data demonstrate that the improved thresholds for NC and BC compared with CC are explained by the increased use of adaptive strategies, i.e., stronger reliance on auditory information. In other words, our data suggest that manipulating vision nudged participants toward adaptive search strategies, indirectly encouraged them to rely more on the auditory gradient and, in turn, improved frequency discrimination performance. The highly comparable results achieved in the blind condition underline the consequences of this nudging effect.

To confirm this, we examined individual participant data. Specifically, we assessed to what extent the discrimination thresholds of individual participants improved by nudging/forcing (visual conditions NC and BC, respectively) them to rely less on visual information. We next examined whether the participants’ individual frequency discrimination performance improved in accordance with their relative change in the use of non-adaptive and adaptive strategies.

To assess the extent of this change, we first ranked participants based on their relative use of adaptive strategies across trials (adaptive proportion) in BC (Figures 4A,B). Figure 4A shows the respective LFHs in each condition for each participant. Importantly, individuals showed distinct patterns in the relationship of LFH across visual conditions, with the majority (69%: 11 out of 16 participants) exhibiting the tendency of improved frequency discrimination in NC and BC.

To assess to what extent improvements in LFH were related to the relative magnitude of strategy shifts, we next analyzed the change in LFH between the visually impaired and the control condition (NC-CC, BC-CC) as a function of the change in proportions of adaptive strategy use between the respective visual conditions (Figure 4C). This revealed that LFH changes correlated with the magnitude of strategy shifts: participants experienced larger improvements in frequency discrimination for larger proportion shifts from non-adaptive to adaptive strategies. This correlation was highly significant for comparisons between BC and CC, and also detectable for NC-CC. Thus, reducing the informational value of the visual input tended to have a similar effect as eliminating visual information, suggesting that the participants were nudged to rely more on auditory information under the NC condition.

Since all participants completed the visual conditions in the same fixed order—first CC, then NC, and finally BC—the improved performance in NC and BC could potentially be confounded by general learning effects that enhance frequency discrimination ability through practice across trials. To examine this, we compared auditory performance across the first ten and last ten trials within each visual condition in the gradient task (Supplementary Figure 1; PCC = 0.054, PNC = 0.623, PBC = 0.582). This analysis suggested a slight improvement tendency within CC. However, comparing LFH based only on trials from the latter half of CC and BC (*p* = 0.0294) confirmed that any potential unspecific performance improvements do not confound the main strategy choice effect. Crucially, participants received no feedback at any point during the experiment regarding the correctness of their responses or any other task-relevant information. This absence of feedback minimizes the likelihood of performance effects driven by training. Additionally, no systematic improvement was observed across visual conditions within strategy classes (Figure 3C). Performance remained consistent when a particular strategy was adopted, even across different conditions. Taken together with the correlation between adaptive strategy adoption and LFH performance (Figure 4C), these findings strongly suggest that strategy choice is the driver of frequency discrimination performance.

Overall, these findings suggest that the improvement in overall performance under compromised visual input stems not from the altered visual input itself, but from a shift toward adaptive strategies induced by the visual manipulation (Figures 3C, 4C). Participants increasingly relied on these strategies when the use of visual information was impeded, leading to better individual and overall outcomes. However, within each strategy, performance remained constant across conditions, underscoring that adaptive strategy use—rather than visual condition—was the key driver of improved performance. Together, this suggests that flexibly adapting to the sensory landscape by shifting active sensing strategies plays a crucial role in enhancing performance.

## Discussion

This study explored the effects of sensory interactions between vision and audition during unrestricted self-motion. Specifically, our aim was to determine to what extent the presence of visual information affects auditory discrimination acuity and whether the participants’ perceptual thresholds are altered when nudging their choice of navigational search strategy by restricting visual information. We demonstrate that auditory frequency discrimination threshold differences are associated with nudging-induced strategy changes.

The results show that frequency discrimination thresholds improved when participants had restricted visual input. This supports the notion that auditory system sensitivity can be heightened in complex scenarios, especially when vision—typically the dominant sensory modality in spatial navigation (Colavita, 1974; Spence et al., 2012)—is impaired or unreliable. These findings go beyond reports that attribute improved performance in similar tasks primarily to the removal of visual distractions (Posner et al., 1976; Keller and Sekuler, 2015). Rather, the absence of informative visual input encouraged participants to adopt adaptive strategies that capitalized on the auditory feedback provided, leading to improved task performance. This pattern aligns with the Colavita effect, which underscores visual dominance in multisensory integration, especially in spatial tasks. However, when vision becomes less reliable for task-specific goals, the reliance on auditory cues becomes essential, emphasizing the flexibility of sensory systems in adapting to environmental demands, consistent with the adaptive behavioral theory (Barlow, 1961). The fact that the provided auditory stimulus is the sole available cue that allows for ‘successful’ completion of the task—namely localization of the target island—further underlines this argumentation. Our findings suggest that the absence of dominant but task-unrelated visual input not only reduces interference but also fosters conditions that enhance the effective use of auditory information. This highlights the critical role of the quality and relevance of sensory cues in shaping frequency discrimination performance, reinforcing the adaptive behavioral framework wherein individuals dynamically adjust strategies depending on the quantity and quality of sensory information available.

Our data also align with prior research demonstrating that sensory overlap may create competition between modalities, diluting the performance benefits associated with specific tasks (Spence and Driver, 2004; Lavie, 2005; Talsma et al., 2007). Our study builds on this by suggesting that when visual navigational support is impaired, reduced interference from visual input can indeed nudge participants toward heightened reliance on auditory information essential for the task. This nudging effect can be viewed as cognitive reorientation, where participants are compelled to abandon their default reliance on vision, allowing for a more flexible strategy that maximizes the use of remaining, reliable sensory information. This also aligns with the principles of perceptual load theory, which posits that when perceptual resources are fully allocated to the most informative modality, performance improves (Lavie, 1995; Lavie et al., 2004; Murphy et al., 2017).

Together, these findings help pave the way to new approaches in rehabilitation for individuals with unisensory deficits. For instance, people with hearing impairments could benefit from interventions that momentarily reduce their reliance on visual cues, thereby “nudging” them to tap into their residual auditory abilities more effectively.

SITh provides an interactive, naturalistic approach to auditory training that differs from conventional methods, which often rely on structured, repetitive tasks. Our findings suggest that limiting visual input may foster sensory reweighting and promote reliance on auditory cues. The closed-loop design of SITh, where participants’ actions directly shape sensory feedback, enhances engagement and intuitiveness. This framework could be particularly relevant for cochlear implant users, as effective auditory training often requires strategies that facilitate generalization to real-world listening. While further research is needed, SITh’s active and ecologically valid design may offer a promising complement to existing hearing rehabilitation programs by supporting adaptive listening strategies in dynamic environments.

Ultimately, this study underscores the adaptive nature of sensory systems in real-world settings. Rather than being strictly hierarchical, our results demonstrate that sensory processing is adaptable and can rapidly shift according to the environmental context and the availability of reliable sensory inputs. Through selective sensory constraint, individuals may be guided to use sensory information in ways that reveal untapped perceptual potential, leading to improved outcomes in tasks where one sense may otherwise dominate.

## Data Availability

The raw data supporting the conclusions of this article will be made available by the authors, without undue reservation.

## Supplementary material

Supplementary Figure 1 Testing training effects within visual conditions. Each panel depicts data from the three gradient conditions, only for the gradient task. First Ten refers to trials 1 to 10, and Last Ten to trials 21 to 30 of each participant in the tested condition.
