# Supplementary figures and images for: Visual nudging of navigation strategies improves frequency discrimination during auditory-guided locomotion
Source: Front Neurosci. 2025 Mar 19;19:1535759. doi: 10.3389/fnins.2025.1535759 (PMC11963732; doi:10.3389/fnins.2025.1535759)

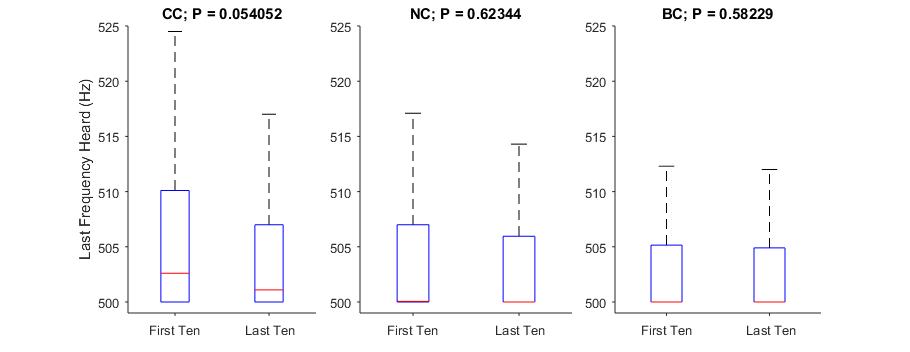

Supplement: Supplementary Figure 1 — Testing training effects within visual conditions. Each panel depicts data from the three gradient conditions, only for the gradient task. First Ten refers to trials 1 to 10, and Last Ten to trials 21 to 30 of each participant in the tested condition. [file Image_1.jpeg]
